# Probable contribution of *Culex quinquefasciatus* mosquitoes to the circulation of chikungunya virus during an outbreak in Mombasa County, Kenya, 2017–2018

**DOI:** 10.1186/s13071-021-04632-6

**Published:** 2021-03-05

**Authors:** Joel Lutomiah, Francis Mulwa, James Mutisya, Edith Koskei, Solomon Langat, Albert Nyunja, Hellen Koka, Samson Konongoi, Edith Chepkorir, Victor Ofula, Samuel Owaka, Fredrick Eyase, Rosemary Sang

**Affiliations:** 1 Kenya Medical Research Institute, Off Mbagathi Way, P.O. Box 54840-00100, Nairobi, Kenya; 2 USAMRU-K, Village Market, P.O. Box 606-00621, Nairobi, Kenya; 3 Institute of Biotechnology Research, JKUAT, P.O. Box 62000-00200, Nairobi, Kenya

**Keywords:** Chikungunya virus, *Culex quinquefasciatus*, *Aedes aegypti*, *Ae. vittatus*, Vector competence

## Abstract

**Background:**

Chikungunya virus is an alphavirus, primarily transmitted by *Aedes aegypti* and *Ae. albopictus*. In late 2017–2018, an outbreak of chikungunya occurred in Mombasa county, Kenya, and investigations were conducted to establish associated entomological risk factors.

**Methods:**

Homes were stratified and water-filled containers inspected for immature *Ae. aegypti*, and larval indices were calculated. Adult mosquitoes were collected in the same homesteads using BG-Sentinel and CDC light traps and screened for chikungunya virus. Experiments were also conducted to determine the ability of *Culex quinquefasciatus* to transmit chikungunya virus.

**Results:**

One hundred thirty-one houses and 1637 containers were inspected; 48 and 128 of them, respectively, were positive for immature *Ae. aegypti*, with the house index (36.60), container index (7.82) and Breteau index (97.71) recorded. Jerry cans (*n* = 1232; 72.26%) and clay pots (*n* = 2; 0.12%) were the most and least inspected containers, respectively, while drums, the second most commonly sampled (*n* = 249; 15.21%), were highly positive (65.63%) and productive (60%). Tires and jerry cans demonstrated the highest and lowest breeding preference ratios, 11.36 and 0.2, respectively. Over 6900 adult mosquitoes were collected and identified into 15 species comprising *Cx. quinquefasciatus* (*n *= 4492; 65.04%), *Aedes vittatus* (*n* = 1137; 16.46%) and *Ae. aegypti* (*n* = 911; 13.19%) and 2 species groups. Simpson’s dominance and Shannon-Wiener diversity indices of 0.4388 and 1.1942 were recorded, respectively. Chikungunya virus was isolated from pools of *Ae. aegypti* (1) and *Cx. quinquefasciatus* (4), two of which were males. Minimum infection rates of 3.0 and 0.8 were observed for female *Ae. aegypti* and *Cx. quinquefasciatus*, respectively. Between 25 and 31.3% of exposed mosquitoes became infected with CHIKV 7, 14 and 21 days post-exposure. For the experimentally infected *Cx. quinquefasciatus* mosquitoes, between 13 and 40% had the virus disseminated, with 100% transmission being observed among those with disseminated infection.

**Conclusions:**

These results demonstrated high risk of chikungunya transmission for residents in the sampled areas of Mombasa. Transmission data confirmed the probable role played by *Cx. quinquefasciatus* in the outbreak while the role of *Ae. vittatus* in the transmission of chikungunya virus remains unknown.
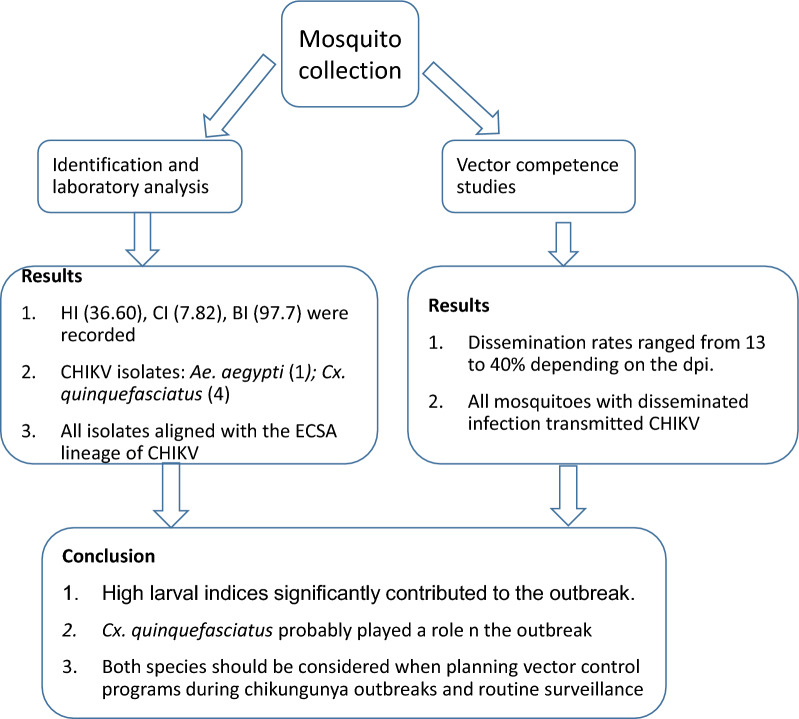

## Introduction

Chikungunya virus (CHIKV) is a global re-emerging mosquito-borne alphavirus, which was first detected in 1952 along the Tanzania-Mozambique border [1]. In Kenya, a chikungunya re-mergence was reported during a major outbreak in 2004–2005 in the coastal region starting in Lamu and spreading to the Indian Ocean islands [2]. This was followed by a huge outbreak in Mandera City, after a lull of 12 years, which affected 10.31% of the population [3], and a year later, another outbreak was reported in Mombasa in 2017/2018.

Historically, CHIKV has been endemic in tropical and subtropical regions of sub-Saharan Africa and Southeast Asia, where two distinct transmission cycles, sylvatic and urban, exist. In Africa, the mosquito species that are traditionally involved in the urban transmission cycle include *Aedes aegypti* and, more recently, *Ae. albopictus* [4]. *Ae. aegypti* is widely distributed in Kenya [5] while *Ae. albopictus* has not been reported. In the sylvatic transmission cycle, a wider range of species is involved including *Ae. aegypti*, *Ae. africanus*, *Ae. luteocephalus*, *Ae. furcifer*, *Ae. taylori, Ae. dalzieli, Ae. metallicus, Ae. neoafricanus, Ae. centropunctatus, Ae. hirsutus, Anopheles domicola, An. funestus, An. coustani, Mansonia uniformis* and *Culex poicilipes* [6–11]. Because of these known traditional vectors involved in the urban transmission cycle of CHIKV, entomological surveillance and outbreak response activities are usually biased toward *Ae. aegypti* or *Ae. albopictus* as the main vectors, leading to neglect of other species even where their densities and possible involvement in the transmission of this virus should be explored. For instance, during chikungunya outbreaks in Reunion and Comoros Islands, CHIKV was isolated from *Culex P. quinquefasciatus* and a pool of *Culex* spp. mosquitoes, respectively, although this was attributed to possible midgut infection and not investigated further [12, 13]. This was probably because species other than *Ae. aegypti* and *Ae. albopictus* have rarely been associated with CHIKV transmission.

Infection with CHIKV is characterized by a spectrum of clinical manifestations ranging from asymptomatic to a mild flu-like syndrome including fever, headache, fatigue, nausea, chills; severe arthralgia and, recently, mortality [14]. These symptoms start 4 to 7 days post-exposure, and most resolve within 2 weeks of the acute phase. However, joint pain can persist for months or years following the initial infection [15].

After laboratory confirmation of human clinical cases during the 2017–2018 outbreak in Mombasa, we investigated entomological risk factors for CHIKV transmission as a means of identifying opportunities for targeted control. We evaluated adult vector occurrence and abundance as well as larval indices and screened the mosquitoes for virus presence. Following the unexpected isolation of CHIKV from *Cx. quinquefasciatus*, we investigated further the ability of this species to amplify and transmit the virus.

## Materials and methods

### Study site description

**Fig. 1:**
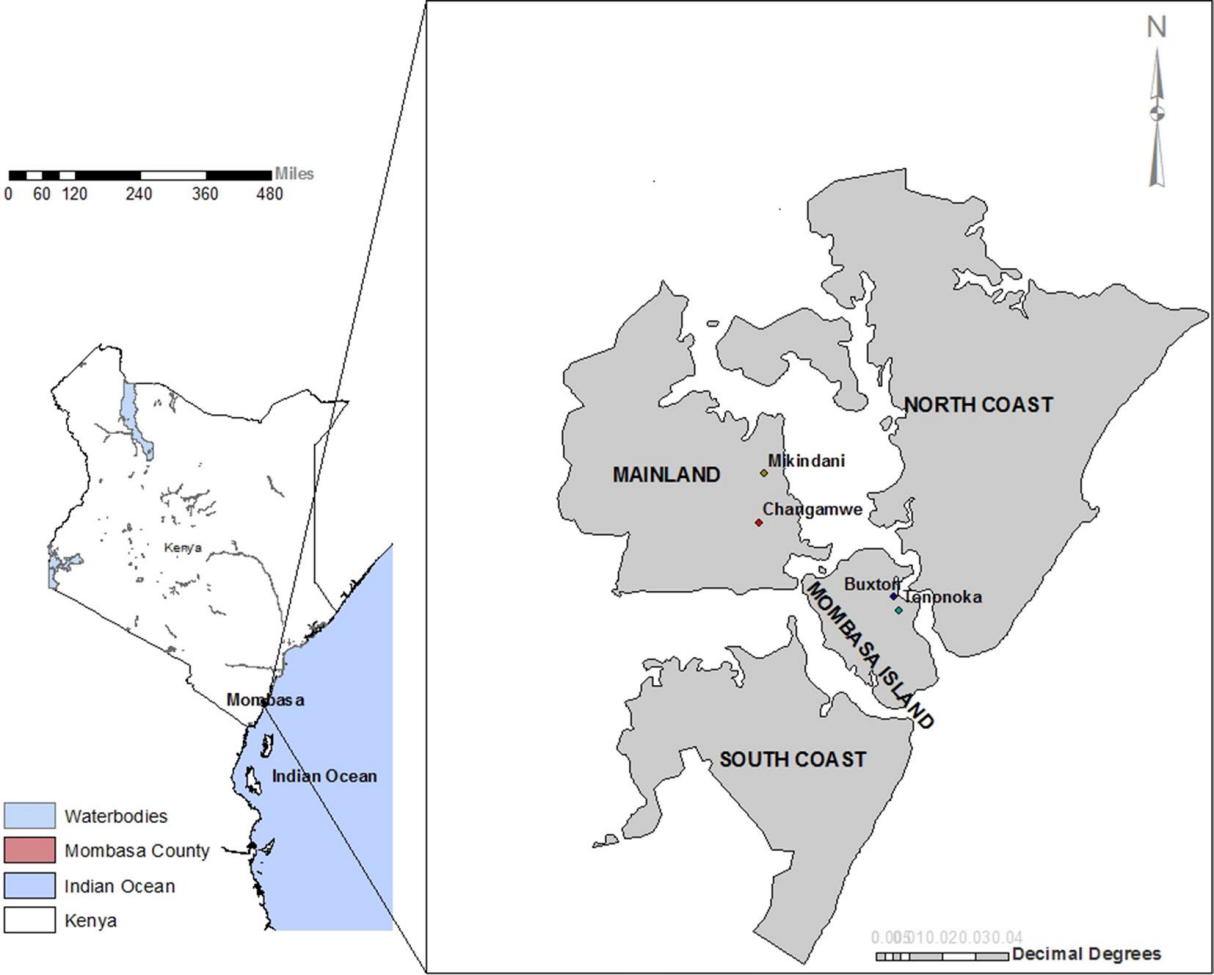
The map of Mombasa county showing outbreak sites where vector sampling was conducted

Mombasa, the study site, is well described by Lutomiah et al. [5]. It is administratively divided into six sub-counties: Changamwe (mainland), Likoni (South coast), Kisauni, Jomvu and Nyali (north coast) and Mvita (Mombasa Island). While chikungunya cases were reported in all six sub-counties, we only conducted sampling in Changamwe (Miritini ward), Mvita (Tononoka ward) and Jomvu (Mikindani ward), which recorded the most cases (Fig. 1).

### Weather data

**Fig. 2:**
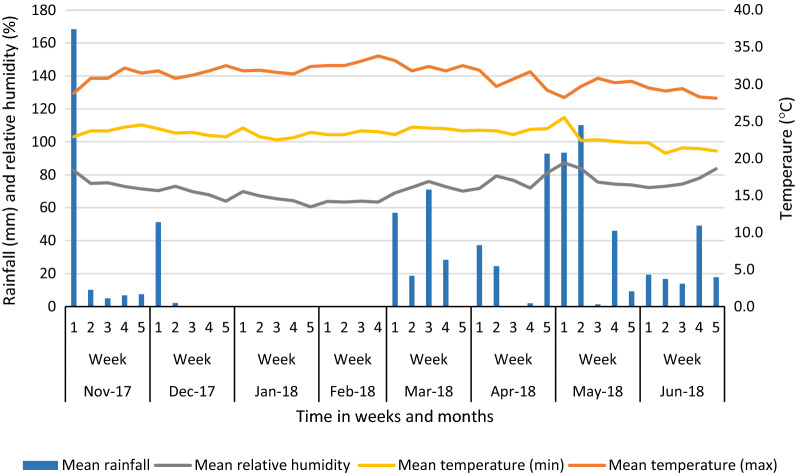
Rainfall, temperature and relative humidity for Mombasa county during the chikungunya outbreak (Source: Kenya Meteorological Department, 2017–2018)

The prevailing weather data for Mombasa during the outbreak were obtained from the Kenya Meteorological Department. Most of December 2017, except for the first week, and January and February 2018 were relatively dry with the lowest mean humidity (67.8%) and mean temperature of 27.8 °C. Subsequently, March to June 2018 were wet months with May receiving the highest amount of rainfall, > 260 mm, and recording the highest mean humidity of 79% (Fig. 2).

### Epidemiological data

An outbreak of chikungunya fever was first reported in Mombasa county, endemic to both dengue and chikungunya, in mid-December 2017. This overlapped with the dengue outbreak that had persisted in the area since April of the same year. The first suspected human clinical cases were reported on 6 December 2017 and confirmed as CHIKV infection on 14 December 2017 at the Arbovirus and Viral Hemorrhagic Fevers (VHF) laboratory at KEMRI’s Centre for Virus Research (CVR). Apparently three of the initial six clinical samples were positive for both DENV and CHIKV IgM, thus indicating co-infections. In total, 19 CHIKV-positive cases were reported in the month of December 2017. The peak of the chikungunya outbreak was in January 2018, and between this month and May 2018, a further 140 positive samples were recorded, mostly in Mvita sub-county. Two of these were co-infected with CHIKV and DENV.

### Collection and rearing of immature mosquitoes from containers in the environment

Entomological surveillance was conducted during the month of January 2018. This coincided with the period when the highest number (*n* = 100) of CHIKV-positive cases was reported. The immatures (larvae and pupae) of *Ae. aegypti* mosquitoes were collected indoors and outdoors over a 10-day sampling period in the areas where chikungunya cases were confirmed. Sampling followed the verbal/oral consent of the household head in the presence of the area headman or sub-chief. All indoor sampling was conducted by field workers accompanied by a household member while outdoor sampling was in the presence of a headman or sub-chief. Individual houses were georeferenced and the demographic data (number of households that spent the night in each house prior to the sampling) recorded. All water-holding containers occurring indoors and outdoors were inspected. Wet containers were scored as either negative (with no *Ae. aegypti* immatures) or positive (with at least one immature *Ae. aegypti*). All immatures from each positive container were collected using white ladles or Pasteur pipettes depending on the container type. For jerry cans, the water was first screened with the help of a flashlight, and if found positive, the water was poured through a fine sieve into a clean white basin. All the immatures were then picked from the sieve immersed in water in the basin using a Pasteur pipette. The type of breeding habitats and their locations were recorded. Live immatures from each container type were put in a whirl pack with information on container type, area/site, whether indoor or outdoor and date of collection and transported in a cool box to the field laboratory. Pupae were separated from larvae, put in pupal cups and then placed in mosquito-rearing cages to develop to adults. Larvae were placed in white enamel trays and fed on fish food (Tetramin^®^) until pupation. The emerging adults were cryo-preserved in liquid nitrogen (LN_2_) for transportation to the VHF laboratory for identification using standard taxonomic keys [16].

### Collection and identification of adult mosquitoes

Adult mosquitoes were collected in the areas where chikungunya cases were confirmed using CDC light and BG-Sentinel (Biogent) traps (John W. Hock). Ten of each of the two trap types were randomly placed in pairs outdoors per day/night, and each was baited with 0.5 kg of dry ice held in igloos next to it [17]. Each pair of CDC and BG-Sentinel traps, approximately 5 m apart, was positioned between 40 and 60 m away from the next pair. In total, 200 traps were set over the 10-day sampling period from 7 to 16 January 2018. The CDC light traps were set at 17:00 and retrieved at 06:00 for 10 consecutive sampling days. The BG-Sentinel traps were set from 17:00 to 17:00 the following day after a 24-h cycle. However, dry ice and collection bags were replaced every morning to target day-biting mosquitoes. All trapped mosquitoes were taken to the site laboratory for sorting and preservation and later transported to the VHF laboratory for identification [16] and pooling (maximum 25/pool) by area/site, sex, species, blood feeding status and date of collection.

### Mosquito processing and virus isolation

Mosquito pools were homogenized using minimum essential medium (MEM) homogenization media supplemented with 15% heat-inactivated fetal bovine serum (FBS) (Sigma-Aldrich), 2% l-glutamine (Sigma-Aldrich) and 2% antibiotic/antimycotic solution (Sigma-Aldrich) with 10,000 U penicillin, 10 mg streptomycin and 25 μg amphotericin B per milliliter. The homogenates were centrifuged at 12,000 rpm for 10 min and the supernatants and pellets stored separately. Fifty microliters each of the mosquito homogenates was inoculated per well in 24-well plates containing confluent monolayers of Vero cells (CCL-81™) (ATCC^®^), grown in MEM (Sigma-Aldrich) growth media, with Earle’s salts and reduced NaHCO_3_, supplemented with 10% FBS, 2% l-glutamine and 2% antibiotic/antimycotic solution. The inoculated plates were incubated at 37 °C for 1 h to allow for virus adsorption and maintained in MEM with 2% FBS, 2% l-glutamine and 2% antibiotic/antimycotic solution at 37 °C. The cells were observed for cytopathic effect (CPE) over a 14-day period and the virus harvested when 75% CPE was observed. Cultures presenting with CPE were passaged to confirm infection and harvested for downstream PCR analysis and sequencing.

### Total RNA extraction and virus identification by reverse transcription PCR

Total RNA was extracted from the supernatants of cell cultures exhibiting CPE using the Qiagen kit following the manufacturer’s protocol. The RNA extract was transcribed into cDNA using the First Strand cDNA synthesis kit (Invitrogen) and random hexamers followed by PCR amplification with Amplitaq Gold PCR master mix (Applied Biosystems) using a panel of primers targeting flavivirus, orthobunyavirus and alphavirus genera. The samples which tested positive with the alphavirus primers were further analyzed using specific primers for CHIKV, ndumu (NDUV), babanki (BBKV) and sindbis (SINV) (Table 1). Amplification products were resolved in 1.5% agarose gel in Tris-borate-EDTA buffer stained with ethidium bromide.

**Table 1:** Sequences and target regions of the primers used in the identification of CHIKV isolates

	Virus or genus	Gene target	Primer sequence	Position	References
1	Flavivirus	NS5	FU 1; (5ʹ-TAC AAC ATG ATG GGA AAG AGA GAG AA-3ʹ)	9007–9032	[18]
		NS5	CFD2; (5ʹ-GTG TCC CAG CCG GCG GTG TCA TCA GC-3ʹ)	9308–9283	
2	Bunyavirus	Nucleocapsid protein	BCS82C; (5ʹ-ATG ACT GAG TTG GAG TTT CAT GAT GTC GC-3ʹ)	86–114	[19]
		Nucleocapsid protein	BCS332V; (5ʹ-TGT TCC TGT TGC CAG GAA AAT-3ʹ)	309–329	
3	Alphavirus	NSP4	VIR 2052F; (5ʹ-TGG CGC TAT GAT GAA ATC TGG AAT GTT-3ʹ)	6917–6997	[20]
		NSP4	VIR 2052R; (5ʹ-TAC GAT GTT GTC GTC GCC GAT GAA3ʹ)	7086–7109	
4	Ndumu	Envelope (E1) gene	ND 124F; (5ʹ-CAC CCT AAA AGT GAC GTT-3ʹ)	124–141	[18]
		Envelope (E1) gene	ND 615R; (5ʹ-ATT GCA GAT GGG ATA CCG-3ʹ)	615–632	
5	Babanki	E1 envelope glycoprotein	Bab 3368F; (5ʹ-CAG CAG ATT GCG CGA CTG ACC-3ʹ)	3368–3388	[18]
		E1 envelope glycoprotein	Bab 4203R; (5ʹ-GCT CAC GAT ATG GTC AGC AGG-3ʹ)	4184–4203	
6	Sindbis	Nonstructural protein	SINV1; (5ʹ-TTT AGC GGA TCG GAC AAT TC-3ʹ)	5194–5213	[18]
		Nonstructural protein	SINV2; (5ʹ-GCG GTG ACG AAC TCA GTA G-3ʹ)	6482–6500	
7	Chikungunya	Capsid gene	CHIK 7028F; (5ʹ-TGC GCG GCC TTC ATC GGC GAC TAC-3ʹ)		[21]
			CHIK 8288r; (5ʹ-CCA GGT CAC CAC CGA GAG GG-3ʹ)		

### Sequencing and phylogenetic analysis

PCR products of the five isolates were purified using the DNA Clean & Concentrator Kit (ZymoResearch, US), and the clean products were sequenced using the Sanger method (Macrogen, Korea). Chromatogram files of the three successfully sequenced isolates were imported into Chromas v2.6, and low-quality regions for each of the forward and reverse reads were trimmed independently and a consensus sequence generated based on the forward and reverse sequences for each of the samples. Sequences generated were compared to those available in Genbank using Blast and phylogenetic analysis was performed using a set of reference sequences obtained from Genbank. Sequences generated were submitted to Genbank under the following ccession numbers: MT992066, MT992067 and MT992068.

### Vector competence studies

Isolation of virus from mosquitoes is not indicative of the mosquitoes being a vector. Therefore, following the isolation of CHIKV from *Cx. quinquefasciatus* mosquitoes, we conducted studies to determine the susceptibility and transmission potential of this species for the virus using an isolate from human serum during the outbreak for all the infection assays. The virus was passaged once in a confluent monolayer of Vero cells in a T-25 cell culture flask, grown in MEM growth media and quantified by plaque assay. The virus was tenfold serial diluted, and the first five dilutions inoculated in 6-well plates, 100 μl/well, containing confluent Vero monolayers as described [22]. The infected cells were maintained with 2.5% methylcellulose mixed with 2× MEM (GIBCO), further incubated at 37 °C for 4 days and then fixed for 1 h with 10% formalin, stained for 2 h with 0.5% crystal violet, washed and the plaques counted to determine plaque-forming units per milliliter (PFU/ml), using the following formula:

$$\frac{{\text{Number of plaques}}}{d \times V} = {\text{ PFU/ml}}$$

where *d* is the dilution factor and *V* is the volume of diluted virus added to the wells [23].

### Mosquito rearing and identification

Immature *Culex* mosquitoes collected from Mombasa were reared in the insectary under standard conditions of 28 °C temperature and 80% relative humidity, with a 12:12-h (light:dark) photoperiod. Adult mosquitoes were inactivated at – 20 °C for 45 s and morphologically identified using the identification key [16] under a dissecting microscope to select *Cx. quinquefasciatus* for use in the study. A leg was also detached from each for subsequent molecular confirmation of species as described by Smith and Fonseca [24]. The mosquitoes were then fed on anaesthetized clean laboratory mice for 45 min to stimulate egg production and provided with an oviposition cup, and the eggs collected were hatched to first filial generation (*F*_1_) for experimental studies.

### Oral infection of *Cx. quinquefasciatus* mosquitoes

Three-to-four-day-old *Cx. quinquefasciatus* females (F_1_) were deprived of glucose for 24 h. The mosquitoes were allowed to feed for 1 h in batches of 50–100 on CHIKV-infectious bloodmeal (log10^8.6^ PFU/ml, determined after 1 h of feeding) in a Hemotek artificial feeding system maintained at 37 °C. Those engorged were transferred to 1-l plastic cages (15–30/cage) with screened tops and maintained on 10% glucose at standard insectary conditions and a 12:12 h light:dark (L:D) photoperiod. The non-engorged mosquitoes were killed by freezing and subsequently incinerated.

### Manipulation of the infected mosquitoes for virus analysis

At 7, 14 and 21 days post-exposure (dpe), 30% of the exposed mosquitoes were picked and sucrose-starved for 16 h. The mosquitoes were cold anesthetized, the legs and wings of each individual mosquito removed using different forceps and pins to avoid cross contamination and placed in the same 1.5-ml microfuge tubes (Eppendorf). The bodies were placed on a sticky tape and proboscises individually inserted into capillary tubes each containing 10–20 µl homogenization media; they were allowed to expectorate for 30 min. The media containing salivary expectorate was expelled into a cryovial with 200 µl of MEM homogenization media. Bodies of mosquitoes, with heads still attached, were each placed in a labeled 1.5-ml microcentrifuge tube containing 1000 μl of MEM homogenization media and homogenized using a mini bead beater (BioSpec Products Inc., Bartlesville, OK, USA) with the aid of copper beads (BB-caliber airgun shot). The homogenates were centrifuged at 12,000 rpm for 10 min at 4 °C and the supernatants stored in 1.5-ml tubes containing 500 µl of MEM homogenization media.

### Determination of infection and dissemination rates and transmission potential

To determine the infection rates, mosquito body homogenates were inoculated in 24-well plates containing confluent monolayers of Vero cells and incubated at 37 °C in 5% CO_2_ for 1 h to allow for virus adsorption. One milliliter of MEM maintenance media was added and the cultures incubated further for 7 days while observing for CPE. Abdominal supernatant of each positive body was serial diluted (tenfold), and 100 µl of each dilution was inoculated to each of ten wells of the 12-well plate with Vero cells grown in MEM. The remaining two wells were inoculated with homogenized male *Cx. quinquefasciatus* mosquito supernatants as negative controls. The plates were incubated at 37 °C for 1 h with frequent agitation every 15 min to allow for virus adsorption. The infected cell monolayers were then overlaid with 2.5% methylcellulose supplemented with 2% FBS, 2% l-glutamine and 2% antibiotic/antimycotic and further incubated at 37 °C. On day 4, plates were fixed for 1 h with 10% formalin, stained for 2 h with 0.5% crystal violet, washed in running tap water and dried overnight. The plates were observed on a light box, and plaques counted and used to calculate the abdominal viral titer and infection rates. For each positive abdomen, corresponding legs/wings were homogenized and their infection status and viral titers determined as described for the abdomens. This process was also repeated for salivary expectorates corresponding to positive legs to determine the transmission rate and the virus titer in the saliva.

### Statistical analysis

Data on adult mosquito collection were analyzed using Simpson’s dominance index and the Shannon-Wiener diversity index. The data on the *Ae. aegypti* survey were analyzed and calculated in terms of four larval indices, which include the house index (HI), container index (CI), Breteau index (BI) and pupal index (PI) or pupae-per-household index (PHI) using the following formulas [25]:

$${\text{HI}} = \frac{{\text{Number of houses with immature mosquitoes}}}{{\text{Number of inspected houses}}} \times 100$$

$${\text{CI}} = \frac{{\text{Number of containers with immature mosquitoes}}}{{\text{Number of wet containers}}} \times 100$$

$${\text{BI}} = \frac{{\text{Number of containers with immature mosquitoes}}}{{\text{Number of inspected houses}}} \times {1}00$$

$${\text{PHI}}/{\text{PI}} = \frac{{\text{Number of pupae collected}}}{{\text{Total number of inspected houses}}} \times {1}00$$

The pupae-per-person index (PPI) was also calculated as the total number of pupae sampled divided by the total population of the inspected households [26]. The risk of transmission of CHIKV was estimated using the WHO criteria. In this case, in an area where the HI, CI and BI exceed 35, 20 and 50, respectively, the risk of Aedes-borne viruses is considered to be high; at BI between 5 and 50, the density of *Ae. aegypti* is considered to be sufficient to promote an outbreak; at HI, CI and BI of 4, 3 and 5, respectively, it is considered unlikely for Aedes-borne virus transmission to occur [27]. Descriptive analysis was used for the distribution of wet containers and *Ae. aegypti* immatures. Wet containers with any number of larvae or pupae were considered “positive containers” while houses with positive containers were considered as “positive houses.” Prevalence of containers was calculated by dividing the total number of container types by the total number of all containers sampled. Container productivity was calculated as a percentage using the formula: number of immatures that emerged from a particular container type/total immatures that emerged × 100. However, to avoid bias arising from one type of container being more prevalent and therefore being sampled than others, mean container productivity was calculated by dividing the total number of *Ae. aegypti* immatures from a particular container type by the total number of that container type that were sampled (number of immatures from a container type/total number of that container type). The 95% confidence interval for container productivity for each category of container was also calculated [28]. The container preferences of *Ae. aegypti* breeding were assessed by determining the breeding preference ratio (BPR) as described by Kumar et al. [29]. This was calculated by dividing the percentage of positive containers by the percentage of containers sampled. Minimum infection rate (MIR) for each species was calculated as the [number of positive pools/total specimens tested] × 1000. All data collected were analyzed using the statistical software package STATA 13.1 (StataCorp LP, TX, USA). The maximum likelihood (ML) tree of Kenyan CHIKV was generated using the Tamura-Nei substitution model and tested using 10,000 bootstraps in MEGA version 7.0.26.

## Results

### *Aedes aegypti* larval infestation levels in different domestic container types

In total, 1636 containers and 7 container types were inspected indoors and outdoors in Tononoka, with jerry cans being the most abundant (*n* = 1232; 75.17%), followed by drums (*n* = 249; 15.19%), buckets (*n* = 96; 5.86%) and basins (*n* = 41; 2.5%), while clay pots were the least abundant. A total of 128 containers (7.81%) were infested with at least one immature *Ae. aegypti* mosquito. Of these, 65.63% were drums followed by jerry cans (14.84%) while clay pots registered 0.78% of all positive containers. All the inspected containers produced a combined total of 528 immature *Ae. aegypti* mosquitoes, with drums being the most productive cumulatively (*n* = 317; 60%) followed by jerry cans (*n* = 74; 14%) and water tanks (*n* = 62; 11.7%) while clay pots were the least productive (*n* = 1; 0.2%). However, mean container productivity showed that water tanks were the most productive with a mean infestation of 6.2 *Ae. aegypti* mosquitoes per container, tire (3.1), drum (1.3) and basin (1.05). Among the sampled container types, BPR was highest for tires (11.36) followed by clay pots (6.50), water tanks (5.13) and drums (4.31) while least for buckets (0.93) (Table 2).

**Table 2:** Container types, positivity, productivity and BPR for immature *Ae. aegypti* mosquitoes

Container type	No. of containers sampled (%)	No. of positive containers (%)	Container productivity (%)	Mean container productivity	Breeding preference ratio
Basins	41 (2.50)	5 (3.91)	43 (8.1)	1.05	1.56
Drums^a^	249 (15.19)	84 (65.63)	317 (60)	1.3	4.31
Jerry cans	1232 (75.17)	19 (14.84)	74 (14)	0.06	0.20
Buckets	96 (5.86)	7 (5.47)	3 (0.5)	0.03	0.93
Tanks^b^	10 (0.61)	4 (3.13)	62 (11.7)	6.2	5.13
Clay pots	2 (0.12)	1 (0.78)	1 (0.2)	0.5	6.50
Tires	9 (0.55)	8 (6.25)	28 (5.3)	3.1	11.36
Total	1639 (100)	128 (7.81)	528 (100)		

Breeding preference ratio (BPR) = percentage of positive containers divided by the percentage of containers sampled

^a^Metal and plastic (50 ≤ 200 l)

^b^Underground, metal, plastic and concrete (500–2000 l)

### Level of household risk for CHIK transmission

A total of 547 people spent the night in the 131 houses inspected on the eves of the sampling, representing a mean population of 4.18/household. Forty-eight of the houses and 128 out of the 1637 inspected containers were positive for immature *Ae. aegypti*; thus, HI = 36.6, CI = 7.82 and BI = 97.71 were recorded during the outbreak. Seventy pupae were also collected, resulting in PHI/PI of 53.44%, while PPI was 12.80%. According to the WHO, thresholds for risk of outbreak/transmission for CI is > 3, HI > 4 and BI > 5 [27]. Therefore, based on our findings, the risk of chikungunya transmission in Mombasa was very high.

### Diversity of adult mosquito species sampled during the outbreak

A total of 6899 adult mosquitoes were collected by BG-Sentinel and CDC light traps from the three sampled sites, Tononoka (*n* = 4733), Mikindani (*n* = 1638) and Miritini (*n *= 528), in decreasing order of abundance. These were identified into 15 mosquito species and 2 species groups, including *Cx. quinquefasciatus* (*n* = 4492; 65.04%), which was the predominant species followed by *Ae. vittatus* (*n *= 1137; 16.46%), *Ae. aegypti* (*n* = 911; 13.19%) and 14 other species (*n* = 367; 5.31%). The least sampled species included *Ae. simpsoni*, *Anopheles gambiae*, *Cx. poicilipes, Eretmapodites chrysogaster* and *Mansonia uniformis*. As expected, overall, BG-Sentinel traps collected more *Ae. aegyti* mosquitoes (*n* = 838) compared to the CDC light traps (*n* = 73) as well as *Ae. vittatus* (*n* = 1106 and *n* = 31, respectively). However, it was interesting to observe higher numbers of *Cx. quinquefasciatus* caught by BG-Sentinel traps (*n* = 4060) compared to CDC light traps (*n* = 432) (Table 3). Simpson’s dominance index and Shannon-Wiener diversity index of 0.4388 and 1.1942 were also respectively recorded for all the adult mosquitoes collected.

**Table 3:** Mosquito species and total collections by BG-Sentinel and CDC light traps

Species	Mvita (Tononoka)					Jomvu (Mikindani)					Changamwe (Miritini)				
	BG		LT			BG		LT			BG		LT		
	M	F	M	F	Total	M	F	M	F	Total	M	F	M	F	Total
*Ae. aegypti*	480	224	31	29	764	31	32	1	4	68	35	36	0	8	79
*Ae. vittatus*	270	836	1	30	1137	0	0	0	0	0	0	0	0	0	0
*Ae. simpsoni*	0	1	0	0	1	0	0	0	0	0	0	0	0	0	0
*Ae. pembaensis*	0	1	0	1	2	0	7	0	0	7	0	0	0	0	0
*Aedes *spp.	42	15	0	3	60	0	0	0	0	0	0	1	0	1	2
*Ae. tricholabis*	0	3	0	2	5	0	5	0	0	5	0	0	0	1	1
*An. gambie*	0	0	0	0	0	0	0	0	0	0	0	1	0	1	2
*Cx. quinquefasciatus*	1484	1044	39	98	2665	330	903	88	142	1463	39	260	1	64	364
*Cx. vansomereni*	5	1	0	1	7	0	0	0	0	0	0	1	0	0	1
*Cx. annulioris*	0	3	0	1	4	0	10	0	4	14	0	3	0	14	17
*Cx. univittatus*	0	4	0	2	6	0	0	0	3	3	0	0	0	45	45
*Cx. poicilipes*	0	0	0	1	1	0	0	0	0	0	0	0	0	0	0
*Cx. zombaensis*	35	40	0	0	75	43	32	0	0	75	2	9	0	5	16
*Culex *spp.	0	0	0	0	0	0	0	0	0	0	0	0	0	1	1
*Er. chrysogaster*	0	2	0	0	2	1	1	0	0	2	0	0	0	0	0
*Mn. uniformis*	0	1	0	1	2	0	0	0	1	1	0	0	0	0	0
*Mn. africana*	0	2	0	0	2	0	0	0	0	0	0	0	0	0	0
Total	2316	2177	71	169	4733	405	990	89	154	1638	76	311	1	140	528

*F* female, *M* male, *BG* Biogent, *LT* light trap

### Chikungunya virus detection and isolation from sampled adult mosquitoes

From 524 pools of adult mosquitoes processed, CHIKV was isolated from 5, 2 each of female (MSA/S24/3013 and MSA/S24/3031) and male (MSA/S23/2481 and MSA/S24/3010) *Cx. quinquefasciatus* mosquitoes and 1 pool of female *Ae. aegypti* (MSA/S23/2444), all collected in Tononoka. These isolations represented minimum infection rates (MIRs) of 3.0 for female *Ae. aegypti*, 0.8 for female *Cx. quinquefasciatus* and 1.0 for male *Cx. quinquefasciatus* mosquitoes (Table 4).

**Table 4:** Mosquito species collected, CHIKV-positive pools and the MIR

Species	Sex	No. of mosquitoes	% of total mosquitoes	No. of pools	CHIKV-positive pools	MIR
*Ae. aegypti*	F	333	4.82	54	1	3.0
*Ae. aegypti*	M	578	8.38	61	0	–
*Ae. vittatus*	F	866	12.55	54	0	–
*Ae. vittatus*	M	271	3.93	20	0	–
*Ae. simpsoni*	F	1	0.01	1	0	–
*Ae. pembaensis*	F	9	0.13	7	0	–
*Aedes *spp.	F	20	0.29	11	0	–
*Aedes* spp.	M	42	0.61	6	0	–
*Ae. tricholabis*	F	11	0.16	6	0	–
*An. gambie*	F	2	0.03	2	0	–
*Cx. quinquefasciatus*	F	2511	36.4	131	2	0.8
*Cx. quinquefasciatus*	M	1981	28.71	112	2	1.0
*Cx. vansomereni*	F	3	0.04	3	0	–
*Cx. vansomereni*	M	5	0.07	2	0	–
*Cx. annulioris*	F	35	0.51	13	0	–
*Cx. univittatus*	F	54	0.78	5	0	–
*Cx. poicilipes*	F	1	0.01	1	0	–
*Cx. zombaensis*	F	86	1.25	15	0	–
*Cx. zombaensis*	M	80	1.16	11	0	–
*Culex* spp.	F	1	0.01	2	0	–
*Er. chrysogaster*	F	3	0.04	2	0	–
*Er. chrysogaster*	M	1	0.01	1	0	–
*Mn. uniformis*	F	3	0.04	3	0	–
*Mn. africana*	F	2	0.03	2	0	–
Total		6899	100	524	5	–

The minimum infection rate was highest among *Ae. aegypti* mosquitoes and lowest in female *Cx. quinquefasciatus*

*F* female, *M* male, *MIR* minimum infection rate

### Sequencing and phylogenetic analysis

**Fig. 3:**
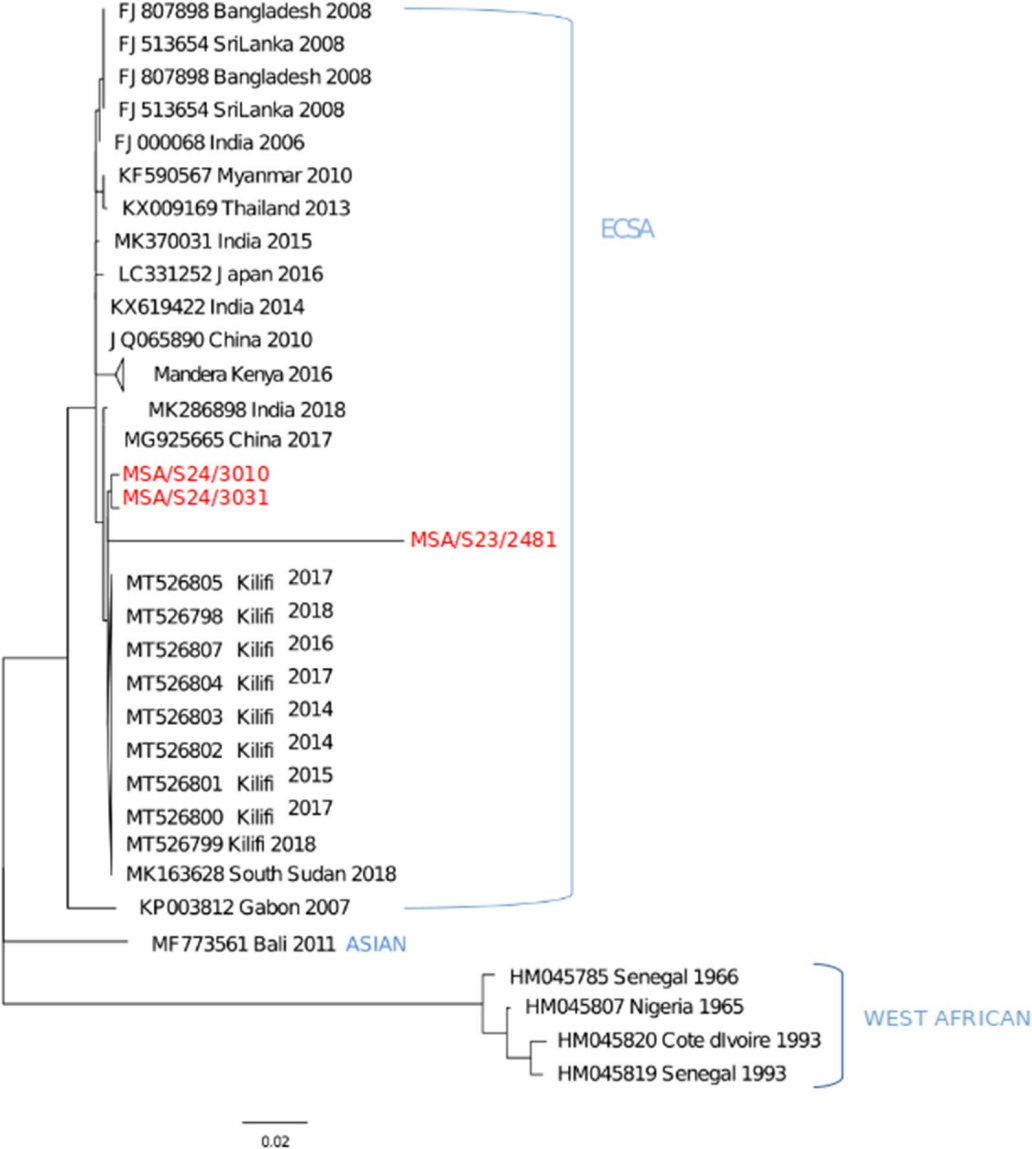
Maximum likelihood phylogenetic tree generated based on sequences belonging to the ECSA, Asian and West African lineages. Sequences obtained in this study are in red fonts

Only three (MSA/S23/2481, MSA/S24/3010 and MSA/S24/3031) out of the five isolates sequenced returned successful sequences. A comparison of the obtained partial sequences showed great similarity with each other. Importantly, they clustered together and formed a clade with isolates previously collected between 2014 and 2018 from Kilifi, a county located in the Southeast Coast region and neighboring Mombasa. These isolates aligned within the ECSA lineage of CHIKV, a clade that also included one isolate from South Sudan (Fig. 3), which neighbors Kenya.

### Susceptibility and transmission rates from vector competence studies

Forty-seven *Cx. quinquefasciatus* mosquitoes were exposed to infectious bloodmeals with viremia of 10^8.6^ PFU/ml. Twenty-five percent of those sampled on day 7 post exposure (dpe) were infected, while 31.3% and 26.7% on 14 and 21 dpe respectively were also infected. The difference in infection rates between 7 and 21 dpe was not statistically significant, Fisher’s exact test = 1.000. Dissemination rates were 25%, 40% and 13.3% on 7, 14 and 21 dpe respectively, with dissemination titers ranging between 10^4.1^ PFU/ml and 10^5.17^ PFU/ml. All the mosquitoes with disseminated infection on 7 and 14 dpe transmitted the virus while there was no transmission on 21 dpe. Transmission titers ranged from 10^5.13^ to 10^5.78^ PFU/ml (Table 5).

**Table 5:** Infection, dissemination and transmission rates for *Cx. quinquefasciatus* mosquitoes after exposure to infectious bloodmeals with viremia of log 10^8.6^ PFU/ml

DPE	No. tested	Infection rate (%)	Titer	Dissemination rate (%)	Titer	Transmission rate^a^ (%)	Titer	Transmission rate^b^ (%)
Day 7	16	4 (25)	nd	1 (25)	4.8	1 (6.3)	5.13	1 (100)
Day 14	16	5 (31.3)	nd	2 (40)	4.1–4.14	2 (12.5)	5.56–5.78	2 (100)
Day 21	15	4 (26.7)	nd	2 (13.3)	4.96–5.17	0	na	na

*nd* not determined

^a^Transmission rate (%) of orally exposed mosquitoes (regardless of their infection status) that transmitted the virus by capillary method

^b^Transmission rate (%) of orally exposed mosquitoes with a disseminated infection that transmitted the virus by capillary method

## Discussion

The global reemergence and spread of CHIKV is driven by the widely distributed primary vector *Ae. aegypti* [30]. There is also evidence that mutations adapt the virus for transmission by new mosquito vectors such as *Ae. albopictus* [31, 32]. Although whether mutations are involved in the transmission of CHIKV by *Cx. quinquefasciatus* has not been determined, control of CHIKV would be best facilitated through identification of breeding habitats of all vectors involved during outbreaks.

Fewer container types were inspected during this outbreak (7) than during the dengue outbreak (17) some years before in the same sites [5]. We attribute this to the dry conditions prevailing during the 2017–2018 sampling period compared to that of 2013–2014, which was during moderate rainfall. Most wet containers were found indoors, and jerry cans were the most frequently sampled (75.26%). However, drums were the most productive containers with 60% of *Ae. aegypti* immatures although they had a moderate BPR of 4.31. While tires had the highest BPR (11.36) and were the second most sampled, jerry cans had the lowest (0.2). The high percentage productivity for drums may be because of their wide openings, which allow easy access for gravid mosquitoes seeking oviposition sites. Most importantly, they also store water for longer, therefore allowing continuous breeding of mosquitoes. Like drums, tires and tanks also remain wet for longer, although their individual productivity during the sampling period was low. For the tires, this was because of the dry spell; hence, only a few of them had water that could support mosquito breeding. Although buckets and basins are used for temporary storage of water in kitchens and/or bathrooms, the water usage practice results in frequent depletion and replenishment, hence not providing sufficient time for mosquitoes to complete the breeding cycle. Traditionally, water tanks are difficult to sample from because of their depth [5], so the observed low numbers of *Ae*. *aegypti* immatures may not reflect their true productivity given that they can retain water for a long time, thus allowing for continuous breeding.

A community’s water storage practice impacts arboviral disease occurrence. Mombasa City authorities continue to face challenges in supplying reliable piped water to its residents. This has influenced the “container-water-storage” practice such that even during the dry season there are wet containers which allow for continuous breeding of *Ae. aegypti*. Hence, the observed larval indices of HI = 36.6%, CI = 7.82% and BI = 97.71% above the minimum thresholds (HI > 4, CI > 3 and BI > 5) for disease transmission [27] may explain the occurrence of chikungunya outbreaks in this region [3]. However, these indices are inadequate to measure transmission risk [33] due to lack of clear correlation with disease transmission [34]. Hence, currently, focus is on the pupal indices as an alternative for assessing the risk of transmission. During this investigation, the PHI/PI = 53.44% and PPI = 12.80% were comparatively higher than those observed in Honduras (0.25) and Brazil (0.15) [35, 36]. Therefore, it is becoming increasingly clear that transmission thresholds are dynamic and differ by geographic regions based on the complexity of factors that influence disease risk and must be determined independently for each region [37–40]. These thresholds have not been determined specifically for Kenya with respect to dengue and chikungunya transmissions. Now with the entry of *Cx. quinquefasciatus* as a competent vector of CHIKV, these indices will play a minimal role in determining risk of disease transmission, so other better means of predicting outbreaks are needed for guiding intervention measures.

Approximately 6900 adult mosquitoes were collected, 911 *Ae. aegypti* and more than 4400 *Cx. quinquefasciatus*. The Simpson’s dominance index of 0.4388 suggested that, at the time, Mombasa County had moderate diversity of mosquito species predominated by *Cx. quinquefasciatus*. The collection of significantly more adult *Ae. vittatus* than *Ae. aegypti* (*P = *0.0001) was also interesting. During previous dengue outbreak investigations in Mombasa and routine surveillance activities in neighboring rural villages, only insignificant numbers were recorded [5, 41] suggesting that this is a recent invasion. This observation, and the sampling of four larvae of this species from drums indoors and tires outdoors, further confirms the changing patterns in their breeding behavior [42] and suggests that the main breeding habitat in Mombasa is unidentified. Traditionally, *Ae. vittatus* breeds in rock pools and tree holes [43] but also in diverse habitats including discarded containers and cement tanks, among others [42, 44, 45].

No CHIKV was isolated from *Ae. vittatus*; hence, its role in the outbreak remains unclear. However, previous experimental studies have shown that they are competent vectors [42, 46, 47]. This species also has an anthropophilic host selection pattern [41, 48, 49]. A combination of these factors would increase its vectorial capacity for CHIKV in urban cycles.

However, CHIKV was isolated from a pool of *Ae. aegypti* and four pools of male and female *Cx. quinquefasciatus* mosquitoes. Traditionally, investigations of chikungunya outbreaks result in few isolates from the primary vector, *Ae. aegypti*. For instance, during the Comoros outbreak investigation, CHIKV was isolated from just two pools of *Ae. aegypti*, despite the magnitude of the epidemic, and one pool of *Culex* spp., although this was attributed to midgut infection and not investigated further [13]. Before this, the virus had also been isolated from *Cx. quinquefasciatus* in Reunion Island but none from *Ae. aegypti* [12]. Some studies have attributed this to inefficient sampling methods for *Ae. aegypti* [50, 51], which are required in large numbers for arbovirus detection [52]. Whereas human landing catch is the most effective, it raises ethical concerns especially during outbreaks due to increased risk of exposure [53]. BG-Sentinel traps, though also considered effective [54], collected only a few (*n* = 333) female *Ae. aegypti* mosquitoes.

Isolation of CHIKV from *Cx. quinquefasciatus* males is the first observation. The main mode of transmitting arboviruses to vectors is through a horizontal process by feeding on viremic hosts. Hence, this observation significantly adds to existing knowledge about vertical transmission playing a role in the natural maintenance of CHIKV in the environment during inter-epidemic periods [55], besides the sylvatic cycle involving mosquitoes, primates and probably rodents. Vertical transmission of CHIKV was previously documented in field-collected and experimentally infected *Ae. aegypti* and *Ae. albopictus* mosquitoes [56–61].

The similarity of the current CHIKV isolates to those found circulating in this region from as early as 2014 [62] suggests that this strain was acquired by *Cx. quinquefasciatus* while feeding on viremic hosts.

Vector competence data incriminated this species as being able to transmit CHIKV. The infectious bloodmeal used had a titer of 10^8.6^ PFU/ml, which is well above the 10^3.6^–10^6.1^ PFU/ml that has been used before to infect *Ae. aegypti* and *Ae. albopictus* mosquitoes [56] but within range (10^4^–10^9^ PFU/ml) of what has been observed in viremic patients [57]. Infection and dissemination rates of *Cx. quinquefasciatus* on 7, 14 and 21 dpe ranged from 25–31% and 13–40%, respectively, while transmission rates on day 7 and 14 dpe were 6.3% and 12.5%, respectively, based on all exposed mosquitoes regardless of infection status, and 100% for those with disseminated infection. This suggests moderate midgut infection (MIB) and escape barriers (MEB) but a weak salivary gland escape barrier (SGEB). However, experimental demonstration of transmission by the capillary method is less efficient than animal models, which are currently unavailable for CHIKV. These experiments confirmed that *Cx. quinquefasciatus* may be contributing to the perpetuation of CHIKV outbreaks that remain unevaluated in the coastal region. Previous experimental studies with *Culex pipiens* showed no evidence of CHIKV transmission [63]. The first laboratory evaluation of vector competence of *Cx. quinquefasciatus* yielded no infections either [8] while subsequent studies showed only midgut infection [64]. Therefore, data from this study add to the increasing spectrum of potential vectors of CHIKV that should be considered in entomological investigations and control strategies during outbreaks.

Other than predominantly feeding on avians [65, 66], *Cx. quinquefasciatus* are also opportunistic feeders and readily feed on humans in urban and peri-urban areas [67]. Therefore, in cities such as Mombasa where this competent species is abundant year-round, *Cx. quinquefasciatus* can contribute to sustained chikungunya outbreaks. Its coexistence with *Ae. aegypti* in the same geographic locales means that when recommending control measures, consideration should incorporate those that target *Cx. quinquefasciatus*.

## Conclusion

We observed high larval indices, which may have significantly contributed to the chikungunya outbreak. The unexpected isolation of CHIKV from *Cx. quinquefasciatus* and the experimental confirmation of transmission of CHIKV point to its possible role in virus circulation in the coastal region. This implies that each outbreak should be approached with an open mind concerning possible incrimination of new vectors involved in the transmission for appropriate vector control measures to be instituted. The coexistence of *Cx. quinquefasciatus* and *Ae. aegypti* in this region will also likely further complicate the vector control processes considering that both species require different approaches. Therefore, these findings can inform future considerations of both species when planning vector control programs during chikungunya outbreaks and routine surveillance activities.

## Data Availability

All data generated or analysed during this study are included in this published article.

## Abbreviations

BBKV: Babanki virus
BG: Biogent
BI: Breteau index
BPR: Breeding preference ratio
CDC: Centers for Disease Control
CHIKV: Chikungunya virus
CI: Container index
CVR: Centre for Virus Research
CPE: Cytopathic effect
DENV: Dengue virus
ECSA: East, Central and Southern Africa
FBS: Fetal bovine serum
HI: House index
LN: Liquid nitrogen
MEM: Minimum essential medium
MIR: Minimum infection rate
NDUV: Ndumu virus
PCR: Polymerase chain reaction
PFU: Plaque-forming units
PHI: Pupae-per-household index
PI: Pupal index
PPI: Pupae-per-person index
RH: Relative humidity
SINV: Sindbis virus
VHF: Arbovirus and viral hemorrhagic fevers
